# RETRACTED: Di Paola et al. Combined Toxicity of Xenobiotics Bisphenol A and Heavy Metals on Zebrafish Embryos (*Danio rerio*). *Toxics* 2021, *9*, 344

**DOI:** 10.3390/toxics13010024

**Published:** 2024-12-30

**Authors:** Davide Di Paola, Fabiano Capparucci, Giovanni Lanteri, Marika Cordaro, Rosalia Crupi, Rosalba Siracusa, Ramona D’Amico, Roberta Fusco, Daniela Impellizzeri, Salvatore Cuzzocrea, Nunziacarla Spanò, Enrico Gugliandolo, Alessio Filippo Peritore

**Affiliations:** 1 Department of Chemical, Biological, Pharmaceutical, and Environmental Science, University of Messina, 98166 Messina, Italy; davide.dipaola@unime.it (D.D.P.); fcapparucci@unime.it (F.C.); glanteri@unime.it (G.L.); rsiracusa@unime.it (R.S.); rdamico@unime.it (R.D.); rfusco@unime.it (R.F.); dimpellizzeri@unime.it (D.I.); aperitore@unime.it (A.F.P.); 2 Department of Biomedical and Dental Sciences and Morphofunctional Imaging, University of Messina, 98100 Messina, Italy; cordarom@unime.it; 3 Department of Veterinary Science, University of Messina, 98100 Messina, Italy; rcrupi@unime.it (R.C.); egugliandolo@unime.it (E.G.); 4 Department of Pharmacological and Physiological Science, Saint Louis University School of Medicine, Saint Louis, MO 63108, USA

The journal retracts the article entitled “Combined Toxicity of Xenobiotics Bisphenol A and Heavy Metals on Zebrafish Embryos (*Danio rerio*)” [1], cited above.

Following publication, concerns were brought to the attention of the Editorial Office regarding the duplication of images between this article [1] and other publications [2,3] produced by the same authorship group.

Adhering to our complaints procedure, an investigation was conducted by the Editorial Office and the Editorial Board that confirmed the duplication of a number of panels within [1] with panels contained within [2,3] presenting different experimental conditions. Following discussions with the authors, the Editorial Board were unable to fully verify the validity of the duplicated images. Consequently, the Editorial Board has lost confidence in the reliability of the findings and decided to retract this publication [1], as per MDPI’s retraction policy (https://www.mdpi.com/ethics#_bookmark30).

This retraction was approved by the Editor-in-Chief of the journal *Toxics*.

The authors did not agree to this retraction.
